# An Analysis of the mRNA Expression of Peripheral-Blood Stem and Progenitor Cell Markers in Pancreatic Neoplastic Disorders

**DOI:** 10.3390/cimb47040236

**Published:** 2025-03-28

**Authors:** Krzysztof Dąbkowski, Maciej Tarnowski, Krzysztof Safranow, Maria Dąbkowska, Alicja Kosiorowska, Kamila Pukacka, Teresa Starzyńska

**Affiliations:** 1 Department of Gastroenterology, Pomeranian Medical University, 71-252 Szczecin, Poland; 2 Department of Physiology in Health Sciences, Pomeranian Medical University, 71-210 Szczecin, Poland; 3 Institute of Physical Culture Sciences, University of Szczecin, 70-453 Szczecin, Poland; 4 Department of Biochemistry and Medical Chemistry, Pomeranian Medical University, 70-899 Szczecin, Poland; 5 Independent Laboratory of Pharmacokinetic and Clinical Pharmacy, Pomeranian Medical University, 70-252 Szczecin, Poland; 6 Department of Pharmaceutical Technology, Pomeranian Medical University, 70-204 Szczecin, Poland

**Keywords:** pancreatic cancer, neuroendocrine tumors, mRNA

## Abstract

Background: Our aim was to assess the expression profiles of the messenger RNA (mRNA) expression profiles of stem-cell genes (*POU5F1*, *NANOG*) and pancreatic progenitor genes (*CK19*, *HES1*, *INS*, *PDX1*) in peripheral-blood mononuclear cells (PBMCs) in selected neoplastic pancreatic diseases, such as cancer and neuroendocrine tumors, to identify neoplastic disease markers in the pancreas. Methods: In this study, 49 patients diagnosed with pancreatic neoplastic diseases (37 with cancer and 12 with neuroendocrine tumors) and 34 control patients, all of whom were hospitalized at a tertiary center, were enrolled. Venous blood samples were collected from the participants, and RNA was extracted from PBMCs. The mRNA expression levels of six stem-cell and pancreatic progenitor markers— *POU5F1* (POU class 5 homeobox 1), *NANOG*, *CK19* (keratin 19), *HES1* (HES family bHLH transcription factor 1), *INS* (insulin), and *PDX1* (pancreatic and duodenal homeobox 1)—were quantified via real-time quantitative PCR. The data were statistically analyzed to explore associations between gene-expression levels and various clinical, biochemical, and morphological parameters (including full blood count, Ca 19-9, weight, height, and BMI) via the Kruskal–Wallis test, Mann–Whitney U test, and Spearman rank correlation coefficient. Results: The results revealed that the expression of the gene associated with early stem cells, *NANOG* (median= 0.002, *p* = 0.03), as well as the genes encoding insulin *INS* (median = 0.004, *p* = 0.02) and *CK19* (median 0.0003, *p* = 0.005), was significantly elevated in patients with pancreatic cancer. However, the gene-expression levels in patients with neuroendocrine tumors did not exhibit statistically significant differences compared to those observed in the control group. Additionally, no significant differences in gene expression were observed among patients at different stages of pancreatic cancer. Furthermore, *CK19* overexpression was found to be positively correlated with inflammatory markers, specifically C-reactive protein (CRP) and WBC, in patients with pancreatic cancer. Conclusions: An elevated mRNA expression of specific stem and pancreatic progenitor genes (*NANOG*, *INS*, *CK19*) in PBMCs may serve as a potential markers for pancreatic cancer, reflecting the disease’s interplay with systemic inflammation.

## Simple Summary

Pancreatic cancer is one of the deadliest cancers, often diagnosed at an advanced stage when treatment options are limited and not effective. This study aimed to identify specific genetic markers in the blood that could help detect pancreatic cancer. We analyzed the expression of six genes in peripheral-blood mononuclear cells from the patients with pancreatic cancer, neuroendocrine tumors, and healthy individuals. The results showed that three genes—*NANOG*, *CK19*, and *INS*—were significantly elevated in patients with pancreatic cancer compared to healthy controls. These genes were not elevated in patients with neuroendocrine tumors, suggesting that this phenomenon may be specific to pancreatic cancer. Additionally, *CK19* was associated with inflammation, a common characteristic of pancreatic cancer. These findings underscore the importance of studying mRNA expression in peripheral blood to enhance our understanding of pancreatic neoplasms and highlight the potential for using blood-based genetic markers to improve the detection of pancreatic cancer. With further validation, this approach could pave the way for less invasive and more accessible diagnostic tools.

## 1. Introduction

Pancreatic ductal adenocarcinoma (PDAC) is a tumor with a rising incidence that constitutes 90% of all pancreatic malignancies and is characterized by intricate interactions between genetic, epigenetic, and microenvironmental factors that contribute to an aggressive nature and late-stage diagnosis, resistance to chemotherapy, and early metastasis [1,2]. It develops from the exocrine part of the gland and is one of the deadliest solid tumors in humans. Despite the development of oncology and diagnostic methods, the prognosis for this malignancy has not changed significantly for years, and no more than 10% of patients survive 5 years from diagnosis. The poor prognosis of patients with PDAC is largely attributable to the complex interplay of factors within the tumor microenvironment (TME), which not only support tumor growth and progression but also contribute to immune evasion and resistance to conventional treatments [3].

The TME in PDAC is characterized by a dense desmoplastic stroma of activated fibroblasts, extracellular matrix components, and many immune cells that collectively create a hostile environment for therapeutic intervention. This stromal barrier is not only a passive system but also an active contributor to tumor biology, as it secretes growth factors, cytokines, and extracellular matrix proteins that promote tumor survival, invasion, and metastasis [4,5]. The stroma also serves as a physical barrier to the delivery of chemotherapeutic agents and increases the difficulty of treatment [6]. Heterogeneity within the tumor microenvironment and differential gene expression have been observed in the tumors of PDAC patients [7,8,9].

In contrast, pancreatic endocrine tumors (PNETs) are less common and often present different clinical characteristics. PNETs can range from benign, well-differentiated tumors to highly aggressive, poorly differentiated forms, which complicates their management due to variability in clinical behavior and the absence of reliable early biomarkers [10,11]. Functional PNETs that secrete hormones can lead to symptomatic presentations associated with hormone hypersecretion, whereas nonfunctional PNETs are often discovered incidentally during imaging for unrelated conditions [12,13].

Peripheral-blood mononuclear cells (PBMCs) have emerged as promising noninvasive biomarkers because of their accessibility and role in reflecting systemic changes associated with cancer. PBMCs, which include lymphocytes and monocytes, are isolated from blood via density gradient centrifugation. This technique allows a diverse array of cells to be obtained from a simple blood draw, thus providing a minimally invasive method for studying gene expression associated with various diseases, including cancer [14]. In pancreatic cancer, PBMCs provide insights into the systemic effects of tumors, particularly in terms of gene expression associated with stem cells and progenitor cells. Cancer stem cells (CSCs) are a type of tumor cell characterized by their ability to self-renew and differentiate into various cell types and play crucial roles in tumor initiation, progression, and resistance to therapy [15,16]. At the cellular level, there has been significant interest in identifying biomarkers that reflect the presence and activity of these cells. Among the most promising candidates are genes related to stemness and progenitor-cell functions, including *NANOG*, *POU5F1*, *CK19*, *HES1*, *PDX1*, and *INS*. These genes are critical not only for maintaining the pluripotency of stem cells but also for their involvement in the pathological processes of tumorigenesis, metastasis, and resistance to therapy [17,18,19]. The alterations of progenitor- and stem-cell genes and their influence on survival and treatment were previously described in different malignancies including inter alia breast, renal cell cancer, lung cancer, and seminoma [20,21,22,23,24].

The development of pancreatic malignancies is related to metabolic alterations [25]. Moreover, genetic alterations in both progenitor genes and stem-cell genes and markers have been described in patients with pancreatic neoplastic and inflammatory disorders [26,27]. The challenge of early detection is underscored by the lack of specific and reliable biomarkers that can differentiate PDAC from other pancreatic conditions, including benign neoplasms and chronic pancreatitis. Currently, the only widely accepted biomarker for pancreatic cancer is carbohydrate antigen 19-9 (Ca 19-9), which is commonly used in clinical practice to monitor disease progression and response to therapy [28]. Ca 19-9 lacks sensitivity and specificity, particularly in the early stages of the disease [29]. As a result, novel biomarkers that can facilitate early diagnosis, predict prognosis, and monitor therapeutic responses in PDAC patients are needed.

*NANOG* and *POU5F1* are well-known transcription factors that play critical roles in maintaining the pluripotency of embryonic stem cells, and their expression has been associated with poor prognosis in various cancers, including PDAC [30]. *NANOG* has been shown to enhance the self-renewal capabilities of CSCs and promote resistance to chemotherapy by activating survival pathways such as the PI3K/Akt and Wnt/β-catenin signaling cascades [31,32]. *POU5F1*, on the other hand, has been implicated in epithelial–mesenchymal transition (EMT), a process that endows cancer cells with enhanced migratory and invasive properties, facilitating metastasis [33]. *CK19*, a cytokeratin typically expressed in epithelial cells, is another marker of interest in PDAC. *CK19* is widely used as a diagnostic marker for PDAC, distinguishing it from other types of pancreatic neoplasms, such as acinar cell carcinoma and neuroendocrine tumors [34]. High *CK19* expression has been correlated with a greater incidence of lymph node metastasis and poorer overall survival, indicating its potential role as a prognostic marker [35]. Moreover, recent studies suggest that *CK19* may play a direct role in the metastatic process, possibly through interactions with the extracellular matrix and modulation of EMT pathways [36].

In addition to these well-established markers, other genes involved in pancreatic development and function, such as *HES1*, *PDX1*, and *INS*, have also been implicated in pancreatic tumorigenesis. *HES1*, which is transcriptionally repressed or regulated by the Notch signaling pathway, plays a crucial role in maintaining the undifferentiated state of pancreatic progenitor cells and has been associated with the suppression of differentiation and promotion of tumorigenesis in PDAC [37]. *PDX1*, another key transcription factor, is essential for the development of the pancreas and the differentiation of pancreatic progenitor cells into insulin-producing beta cells [38]. The dysregulation of *PDX1* has been linked to the development of PDAC, particularly in the context of hyperinsulinemia and insulin resistance, which are recognized risk factors for pancreatic cancer [38]. *INS*, the gene encoding insulin, has also been implicated in the pathogenesis of PDAC, where aberrant insulin signaling and hyperinsulinemia are thought to contribute to the metabolic alterations that support tumor growth and survival [39].

While significant progress has been made in understanding the molecular mechanisms underlying PDAC, the identification of reliable biomarkers for early detection and prognosis remains a critical unmet need.

In our study, we comprehensively assessed the mRNA expression profiles of stem cell- and progenitor-cell-associated genes in peripheral-blood mononuclear cells from patients with different clinical stages of pancreatic malignancies (PDAC and PNET). By analyzing these profiles, we aimed to identify potential biomarkers or biomarker combinations that could facilitate the noninvasive diagnosis, prognosis, and monitoring of pancreatic cancer.

## 2. Materials and Methods

### 2.1. Patients and Study Design

Blood was collected from the forearm veins of patients hospitalized at the Department of Gastroenterology, Pomeranian Medical University (Szczecin, Poland), with pancreatic neoplasms (37 with cancer and 12 with neuroendocrine tumors) in different clinical stages (14 with locally advanced cancer, 23 with metastatic cancer, and 12 with operative neuroendocrine tumors) and from 34 healthy controls. The diagnosis of pancreatic neoplastic disease was confirmed with EUS-guided biopsy, and in every patient, clinical staging was assessed with abdominal computed tomography and chest X-ray. The full blood count, CRP, and Ca 19-9 were measured using standard hospital laboratory tests. The study was approved by the Bioethics Committee of Pomeranian Medical University kB-0012/43/12 (date of approval 19 May 2015). Written informed consent was obtained from all patients.

### 2.2. Assessment of mRNA in Peripheral Blood

Peripheral-blood samples (~20 mL) were collected from forearm veins. Red blood cells were removed from the PB samples via 1X hypotonic lysis solution BD Pharm Lyse Buffer, (BD Bioscience Pharmingen, Franklin Lakes, NJ, USA). Total nucleated cells were washed with Dulbecco’s phosphate-buffered saline (DPBS, w/o Ca^2+^ and Mg^2+^; HyClone, (GE Healthcare Life Sciences, Chicago, IL, USA) and immediately stored in RNAlater^®^ solution (Invitrogen, Carlsbad, CA, USA at –80 °C until genetic analysis. Total RNA was isolated from lysed peripheral blood with an RNeasy Kit (Qiagen, Hilden, Germany. The RNA was reverse-transcribed with a first-strand cDNA synthesis kit and oligo-dT primers (Thermo Fisher, Waltham, MA, USA). The mRNA expression of 6 stem cell and pancreatic progenitor genes, *POU5F1* (POU class 5 homeobox 1), *NANOG*, *CK19* (*keratin 19*), *HES1* (*hes family bHLH transcription factor 1*), *INS* (*insulin*), and *PDX1* (pancreatic and duodenal homeobox 1), was assessed via real-time quantitative PCR on an ABI 7500 Fast (Applied Biosystems, Foster City, CA, USA). The real-time conditions were as follows: 95 °C (15 s), 40 cycles at 95 °C (15 s), and 60 °C (1 min). Melting curves were generated after each reaction to verify the melting temperature of the amplicon and only one PCR product was amplified under these conditions. In order to ensure the consistency of the qPCR measurements each sample was analyzed in two technical replicates, and mean Ct values were used for further analysis. The mRNA expression of all the genes investigated was normalized to that of the nonregulated reference housekeeping gene b-2 microglobulin (*B2M*). The data are presented as absolute expression values via the 2ˆdCt formula. The primers (Oligo.pl, IBB, Warsaw, Poland) used were as follows: *Oct-4* (forward) 5′-CCCCTGGTGCCGTGAA-3′, (reverse) 5′-GCAAATTGCTCGAGTTCTTTCTG-3′; Nanog (forward) 5′-GCAGAAGGCCTCAGCACCTA-3′, (reverse) 5′-AGGTTCCCAGTCGGGTTCA-3′; *CK19* (forward), (reverse); *HES1* (forward) 5′-GCCGCGAGCTATCTTTCTTCA-3′, (reverse) 5′-ACACGACACCGGATAAACCAA-3′; INS (forward) 5′-GCAGCCTTTGTGAACCAACA-3′, (reverse) 5′-TTCCCCGCACACTAGGTAGAGA-3′; and *PDX1* (forward) 5′-AACCGCGTCCAGCTGCCTTTC-3′, (reverse) 5′-CCGCTTGTTCTCCTCCGGCTC-3′.

### 2.3. Statistical Analysis

Since most quantitative variables, including mRNA expression, showed distributions significantly different from a normal distribution, they are presented as medians with interquartile ranges (Q1–Q3), and nonparametric tests were used: the Mann–Whitney U test for comparisons between groups and the Spearman rank correlation rank coefficient (Rs) for correlations within groups. Multivariable analysis comparing PDAC group with controls after adjustment for age and sex (with age, sex and clinical phenotype, i.e., PDAC vs. Controls as independent variables) was performed using general linear model (GLM) with effect size presented as standardized beta coefficient (β). Multivariable stepwise logistic regression with forward selection was applied to find independent markers of PDAC (PDAC vs. Control) in a set of variables containing 6 studied gene-expression levels, CRP, and Ca 19-9 serum concentrations. Gene expressions and CRP or Ca 19-9 concentrations were log-transformed before inclusion into the multivariable analyses to normalize their distributions. Odds ratio (OR) values obtained from logistic regression after such a transformation should be interpreted as an increase in odds for PDAC diagnosis per 2.72 times (natural logarithm base) increase in the corresponding marker expression or concentration. Associations with *p* < 0.05 were considered statistically significant. The statistical power of the study with 37 PDAC patients, 12 PNETs patients, and 34 healthy controls was sufficient to detect with 80% probability true differences between groups corresponding to ±0.68 standard deviations of the studied parameters for a comparison of PDAC vs. controls, and ±0.97 standard deviations of the studied parameters for a comparison of PNETs vs. controls.

## 3. Results

### 3.1. Patient Characteristics

We compared basic clinical data, including age, height, weight, BMI, and biochemical features, such as white blood count and platelets (PLT), erythrocytes (ERY), CRP, and Ca 19-9 counts, between patients with pancreatic cancer and neuroendocrine tumors and healthy controls. Our analysis revealed significantly greater leukocyte counts, CRP and Ca 19-9 levels in PDAC patients and lower weights, BMIs, and erythrocyte counts than in healthy controls. The PNETS patients were significantly younger than the healthy controls were. Table 1 presents the baseline characteristics of all included patients.

### 3.2. Expression of mRNAs Encoding Stem-Cell and Progenitor Pancreatic Genes in PB Patients with Pancreatic Neoplastic Disorders

**Figure 1:**
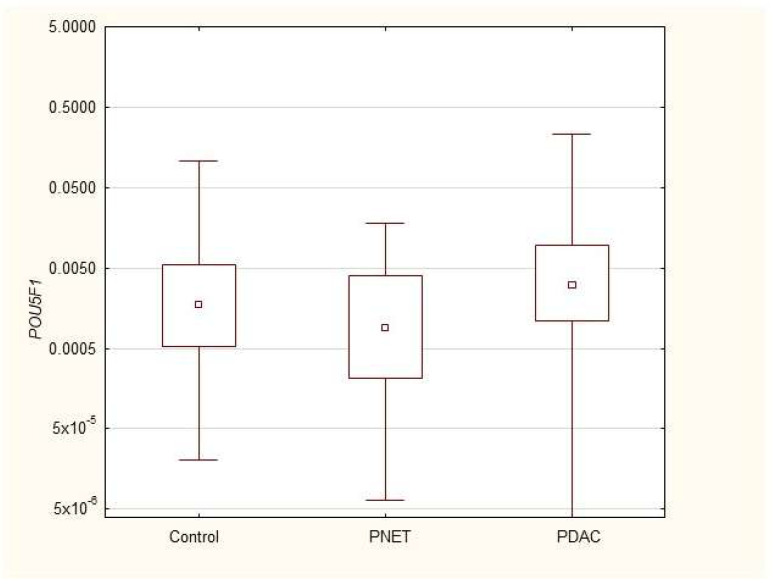
Expression of *POU5F1* mRNA in PDAC, PNET and control patients. Data are presented as medians with interquartile ranges (Q1–Q3), and nonparametric Mann–Whitney U test was used for comparisons between groups. Associations with *p* < 0.05 were considered statistically significant.

**Figure 2:**
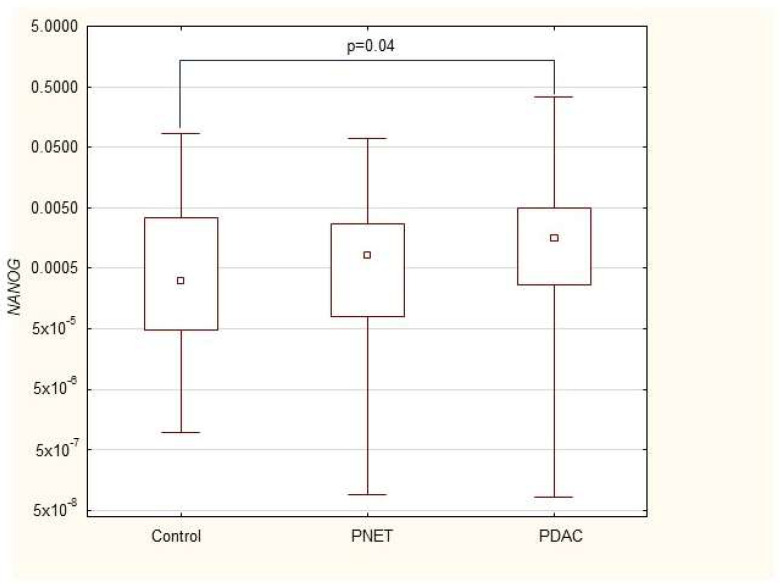
Expression of *NANOG* mRNA in PDAC, PNET and control patients. Data are presented as medians with interquartile ranges (Q1–Q3), and nonparametric Mann–Whitney U test was used for comparisons between groups. Associations with *p* < 0.05 were considered statistically significant.

**Figure 3:**
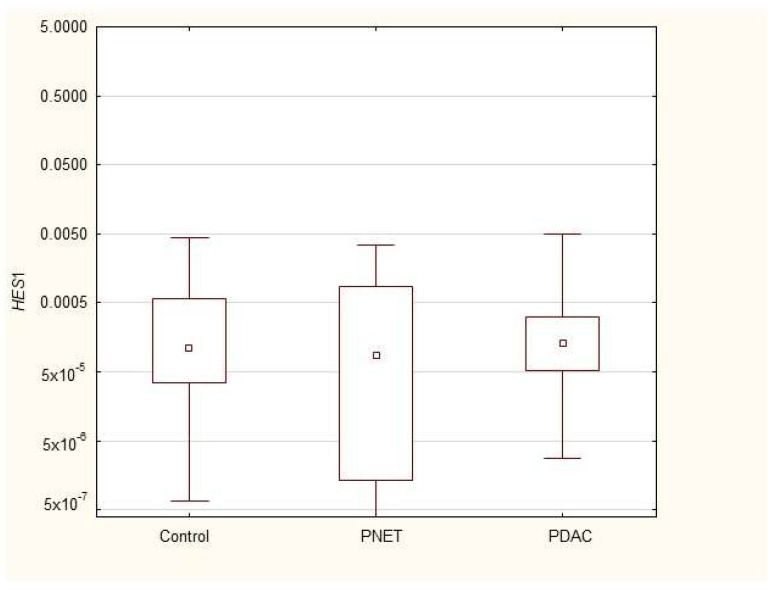
Expression of *HES1* mRNA in PDAC, PNET and control patients. Data are presented as medians with interquartile ranges (Q1–Q3), and nonparametric Mann–Whitney U test was used for comparisons between groups. Associations with *p* < 0.05 were considered statistically significant.

**Figure 4:**
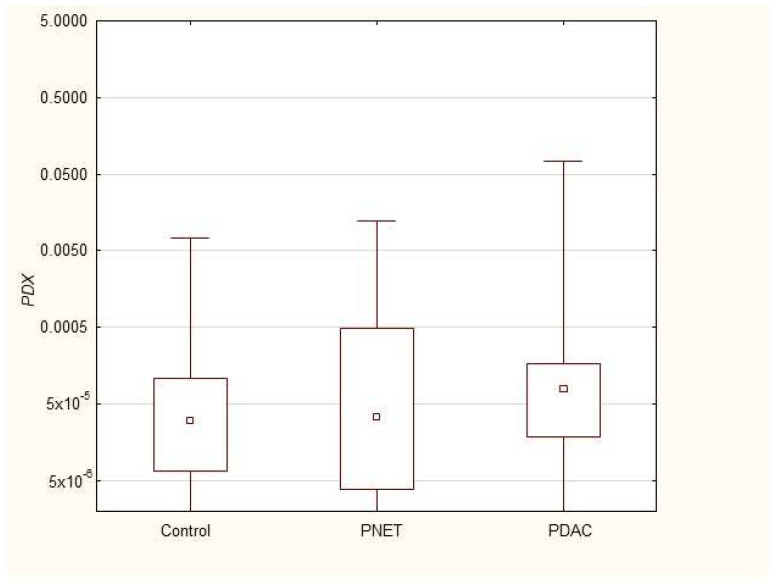
Expression of *PDAC* mRNA in PDAC, PNET and control patients. Data are presented as medians with interquartile ranges (Q1–Q3), and nonparametric Mann–Whitney U test was used for comparisons between groups. Associations with *p* < 0.05 were considered statistically significant.

**Figure 5:**
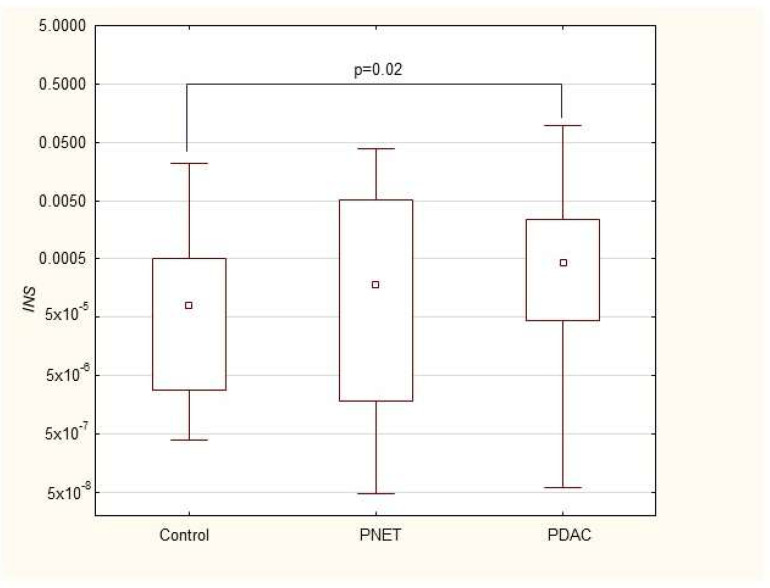
Expression of *INS* mRNA in PDAC, PNET and control patients. Data are presented as medians with interquartile ranges (Q1–Q3), and nonparametric Mann–Whitney U test was used for comparisons between groups. Associations with *p* < 0.05 were considered statistically significant.

**Figure 6:**
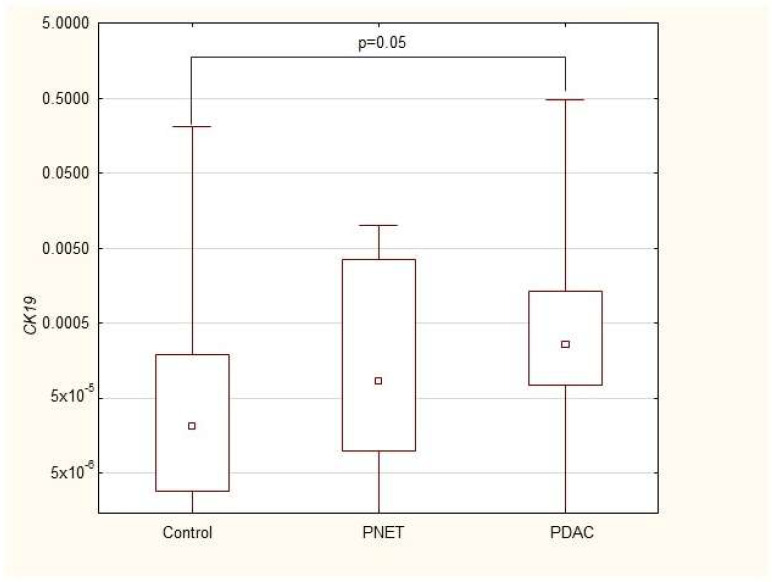
Expression of *CK19* mRNA in PDAC, PNET and control patients. Data are presented as medians with interquartile ranges (Q1–Q3), and nonparametric Mann–Whitney U test was used for comparisons between groups. Associations with *p* < 0.05 were considered statistically significant.

We found that the expression of genes characteristic of early stem cells, *NANOG* (*p* = 0.03), and the expression of genes encoding insulin and cytokeratin 19 (*INS p* = 0.02, *CK19 p* = 0.005) were increased in pancreatic cancer patients. However, it was not significantly different under other neoplastic (neuroendocrine) conditions. Multivariable GLM analysis adjusted for patient age and sex, comparing the PDAC group with controls, showed that the association of PDAC with a higher expression of *CK19* (β = +0.27, *p* = 0.024) and INS (β = +0.23, *p* = 0.048) remained significant, while for *NANOG* (β = +0.20, *p* = 0.093) the association lost significance. No significant collinearity was found between the independent variables (age, sex, and clinical phenotype) in the GLMs. There were no significant differences in genetic expression between patients with different stages of pancreatic cancer (locally advanced and metastatic). Figure 1, Figure 2, Figure 3, Figure 4, Figure 5 and Figure 6 and Table 2 present the expression of genes associated with neoplastic disorders. The gene-expression data and basic clinical characteristics of patients with locally advanced and metastatic pancreatic cancer are presented in Table 3.

### 3.3. Correlations

In pancreatic cancer patients; statistical analysis revealed no significant correlations between the expression of genes and clinical (height; weight; BMI) or biochemical data (WBC; ERY; PLT; CRP; and Ca 19-9); apart from *CK19* overexpression; which was correlated with inflammatory markers (both CRP (Rs = +0.34; *p* = 0.04) and WBC (Rs = +0.51; *p* = 0.001) in patients with pancreatic cancer.

In neuroendocrine tumor patients, statistical analysis revealed no significant correlations between the expression of genes and clinical or biochemical data, with the exception of a positive correlation between *HES1* gene expression and WBCs (Rs = +0.77, *p* = 0.003). The correlations between mRNA gene expression and clinical and biochemical patient characteristics are presented in Supplementary Tables S1 and S2.

### 3.4. Multivariable Analysis of PDAC Markers

Multivariable logistic regression was applied to find independent markers of PDAC among the PBMC expression of studied genes, serum CRP as standard inflammation marker and serum Ca 19-9 as current standard PDAC marker (all log-transformed). When only gene expression values were analyzed, high *CK19* was the best marker of PDAC (OR = 1.19, 95%CI: 1.03–1.39 per 2.72-times increase, *p* = 0.018), and no other gene expression significantly improved the estimation when added to the logistic regression model. When CRP was included, its high concentration proved the best marker of PDAC, and *NANOG* expression was the only gene expression which significantly improved the estimation when added to the model (OR = 2.71, 95%CI: 1.56–4.70, *p* = 0.00032 for CRP and OR = 1.25, 95%CI: 1.01–1.56, *p* = 0.040 for *NANOG* in the two-variable model). The loss of significance of *CK19* as PDAC marker in this model may be attributed to collinearity between CRP and *CK19* (significant positive correlation described above). Finally, when Ca 19-9 was included, it proved the best marker of PDAC (OR = 3.12, 95%CI: 1.80–5.41, *p* = 0.000036), and neither CRP nor any gene expression significantly improved the estimation when added to the model.

## 4. Discussion

Pancreatic cancer continues to be one of the most significant challenges in oncology, with PDAC exhibiting a particularly dismal prognosis due to late-stage diagnosis, resistance to chemotherapy, and a lack of effective biomarkers for early detection. The five-year survival rate remains below 5%, underscoring the urgent need for novel molecular markers that could improve early diagnosis, facilitate risk stratification, and enhance therapeutic decision-making [40].

In this study, we conducted a comprehensive analysis of mRNA expression levels of key progenitor- and stem-cell-associated genes in the peripheral blood of patients with pancreatic neoplastic disorders, including PDAC and PNETs. Our findings revealed a significant overexpression of *NANOG*, *CK19* and *INS* mRNA in the serum of PDAC patients compared to healthy controls, independent of disease stage. In contrast, this pattern was not observed in patients with PNETs, where expression levels of these genes were notably lower.

To our knowledge, no prior studies have investigated the expression of these genes in the serum of patients with PNETs. The observed discrepancies between PDAC and PNETs may be attributed to fundamental biological differences between these malignancies. While PDAC arises from exocrine part of the gland, PNETs originate from neuroendocrine cells of the diffuse endocrine system. The clinical behavior of PNETs is highly heterogenous, ranging from indolent, slow-growing tumors to aggressive subtypes associated with poor outcomes. Furthermore, PNETs are often diagnosed at a younger age and earlier stage than PDAC, with functional PNETs frequently presenting with hormone-related symptoms, as seen in insulinomas or gastrinomas. These biological and clinical distinctions may underlie the differential mRNA expression patterns observed in our study.

The overexpression of stem-cell markers in PDAC patients aligns with our previous findings of increased circulating stem cells in the peripheral blood of patients with pancreatic cancer [27]. *NANOG* is a gene that controls stem-cell pluripotency, and has been implicated in tumor progression, metastatic potential, and poor prognosis in PDAC [41]. However, in our study, no significant difference in *NANOG* expression was observed between patients with locally advanced and metastatic disease. This may be explained by the fact that both metastatic and inoperable locally advanced PDAC cases are associated with extensive tumor angiogenesis and the presence of circulating tumor cells.

The stage of pancreatic cancer can significantly influence mRNA expression profiles in PBMCs due to factors such as tumor burden, immune-response alterations, and systemic inflammation [42]. As the pancreatic cancer, it can modulate the immune responses, further affecting PBMC gene expression [42]. Moreover, late-stage PDAC is often characterized by hypoxia and cellular reprogramming, where hypoxia-inducible factors may alter PBMC mRNA expression [43]. Furthermore, tumors at advanced stages release increased amounts of exosomes, microRNAs, and other soluble mediators that can modify PBMC function and transcriptional activity. The elevated levels of cytokines such as IL-6, TNF-α, and TGF-β in later-stage PDAC further contribute to immune dysregulation, potentially affecting PBMC gene expression and promoting an immunosuppressive microenvironment [44]. These complex interactions between tumor progression, immune modulation, and systemic inflammation also explain the observed overexpression of *NANOG* in PDAC. Our previous observations, along with murine studies, suggest a crucial role of mobilized bone marrow-derived stem cells in supporting the growth metastasis [26,45]. The specificity of *NANOG* as a potential biomarker is further reinforced by its absence in patients with PNETs in our study. This distinction highlights its potential diagnostic use in differentiating PDAC from other pancreatic neoplasms. Furthermore, these findings build upon our earlier research, which showed the critical involvement of pluripotent stem cell in the pathogenesis of pancreatic cancer [27,46].

*CK19* is a type of filament protein expressed in the epithelium of the gastrointestinal tract and is commonly associated with tumors of epithelial origin, including circulating tumor cells in the bloodstream [16]. Its expression has been observed in various types of cancers, including breast, lung, PDAC, and pancreatic neuroendocrine tumors [17,18,19]. *CK19* has been consistently associated with poor prognosis, tumor progression, and metastatic development [16]. In our study, we observed that *CK19* mRNA expression was elevated exclusively in the serum of pancreatic cancer patients and was positively correlated with systemic inflammation, as indicated by increased WBC and CRP levels, two commonly used clinical inflammatory markers. These findings align with the existing literature highlighting that higher CRP concentrations correlate with reduced survival rates in PDAC patients [47,48]. Similarly, increased WBC counts have been linked to tumor progression and reduced survival rates in pancreatic cancer patients [49,50,51]. Moreover, multivariable GLM analysis revealed that *CK19* expression showed the strongest association with PDAC among the studied markers. However, it was also most closely correlated with CRP. Including CRP in the logistic regression model diminished the predictive value of *CK19* for PDAC, suggesting that *CK19* expression largely reflects inflammation severity. Interestingly, adding *NANOG* expression to CRP in the model enhanced PDAC prediction, possibly indicating that elevated *NANOG* expression reflects additional, non-inflammatory mechanisms in PDAC pathology.

The correlation between inflammation, the exosome-mediated signaling, and stem-cell expression was previously demonstrated in PDAC [27,52]. In this group of patients, complement cascade elements (C3a and C5a) play a pivotal role in stem-cell trafficking to the peripheral-blood components [17] and modulate the inflammatory environment of PDAC. They reinforce immune-cell recruitment and stimulate cytokine release, altering the distribution of *CK19*-positive tumor cells as circulating tumor cells [53]. This interaction highlights *CK19*’s dual role as both a marker of epithelial tumors and a component of inflammation-driven tumor progression in PDAC. Moreover, chronic inflammation, a hallmark of PDAC, leads to widespread immune activation, which extends beyond the tumor microenvironment to circulating immune cells, including PBMCs. Inflammatory cytokines and tumor-derived mediators released into the bloodstream interact with PBMCs, triggering intracellular signaling pathways that upregulate *CK19*, *NANOG*, and *INS* genes [52]. A key pathway implicated in this process is the PI3K-Akt signaling cascade, which is frequently activated in cancer. It is crucial in cellular processes’ regulation such as metabolism, growth, and survival. It ultimately leads to alterations in gene-expression patterns in PBMCs [54]. The phosphorylation-mediated inactivation of transcription factors such as FOXO1 may thus represent a mechanistic link between systemic inflammation and the upregulation of *CK19* in circulation immune cells, further reinforcing its role in PDAC pathogenesis.

Additionally, cytokines such as IL-6, IL-1β, and TNF-α, play important roles in cancer initiation, growth, and metastasis development [55,56]. IL-6, for instance, activates the JAK/SSTAT3 signaling pathway, which is critical for promoting *CK19* transcription and sustaining tumor-cell proliferation and invasiveness [57]. IL-6 is also known to increase CRP levels, directly linking *CK19* expression with systemic inflammatory markers such as the WBC count and CRP level [58]. The desmoplastic PDAC microenvironment is complex, and the interplay between its components, including inflammatory elements, determines cancer progression [10]. In addition, TNF-α and IL-1β activate the NF-κB pathway, a master regulator of inflammation and oncogenesis in PDAC, further increasing *CK19* expression. The persistent activation of NF-κB in the PDAC microenvironment results in a feedback loop that amplifies cytokine secretion, driving both tumor progression and the inflammatory response [59]. Moreover, carcinogenesis shares some similarities with the process of inflammation, and inflammation may trigger cancer development [46,60]. This is also observed clinically, including in chronic atrophic gastritis, ulcerative colitis, and chronic pancreatitis, which eventually lead to gastric cancer, colon cancer, and pancreatic cancer, respectively [60,61].

In contrast to previous studies, in which *CK19* expression has been identified as a marker of poor prognosis in PNETs, particularly in advanced or metastatic cases, our study did not observe significant *CK19* expression in patients with PNETs [57]. This may be explained by the characteristics of the patient population included in this study, which consisted primarily of individuals with early-stage, nonmetastatic PNETs. It is possible that *CK19* expression becomes more prominent in more aggressive or advanced disease stages and thus may not be relevant in early tumor presentation. Furthermore, when PNETs are compared with PDAC, *CK19* expression patterns may differ, as PDAC often presents at more advanced stages where poor prognostic markers such as *CK19* are more prevalent. These findings suggest that *CK19* may be more useful as a prognostic biomarker in late-stage PNETs or PDAC patients than in early, localized PNET patients.

*INS* is a fundamental gene involved in glucose metabolism, and its dysregulation has been implicated in the pathogenesis of several cancers, including PDAC. In the present study, we observed significantly elevated mRNA levels of *INS* in PBMCs from patients with PDAC compared to healthy controls. This observation aligns with previous findings that highlight the insulin/IGF signaling axis as frequently dysregulated in PDAC, contributing to both the metabolic reprogramming and stromal remodeling characteristic of this malignancy [62,63,64]. Pancreatic cancer cells secrete exosomes—nano-sized extracellular vesicles encapsulating proteins, lipids, and RNAs that can fuse with PBMCs, delivering their cargo and modulating gene expression. It other studies, it was demonstrated that pancreatic cancer-derived exosomes induce insulin resistance in recipient cells through the PI3/Akt/FOXO1 signaling pathway, highlighting their role in altering cellular function [65]. Hyperinsulinemia and insulin resistance, common features in PDAC, have been demonstrated to promote tumor growth, progression, and the development of a desmoplastic stroma [31,32,39]. The stromal microenvironment actively supports tumor progression through complex interactions between tumor and stromal cells via paracrine signaling pathways involving insulin and IGF-1 receptors. Similarly to the *CK19* and *NANOG*, the elevated expression of *INS* mRNA in PBMCs was statistically significant, but the relative differences between PDAC patients and controls was modest, and the data demonstrated considerable inter-individual variability. This variability highlights the challenges of using PBMC-derived biomarkers in the context of PDAC and suggest that, although *INS* mRNA expression may reflect systemic metabolic changes associated with the disease, it cannot be entirely linked to tumor-specific gene expression in this study. This is in line with previous reports that have shown systemic dysregulation of the insulin/IGF pathway in PDAC, which may not directly mirror the gene-expression profiles within the tumor itself [66]. Instead, these alterations represent a systemic response to tumor-induced metabolic shifts and stromal activation, both of which contribute to the pro-tumorigenic microenvironment.

The tissue degeneration process, involving the replacement of the pancreatic parenchyma with fibrosis or the development of edema or necrosis, followed by the onset of endocrine and exocrine gland insufficiency, forms the core of pancreatitis. Unsurprisingly, disturbances in the expression of genes involved in pancreatic development, such as *HES1* and *PDX1*, were observed in our study. These genes belong to the family of transcriptional activators. *PDX1* plays crucial roles in maintaining B-cell mass, function, and identity, which are essential for the expression of insulin, somatostatin, glucokinase, islet amyloid polypeptide, and glucose transporter type 2 [67]. Its defects are responsible for pancreatic agenesis and the development of diabetes (maturity-onset diabetes of the young type 4-MODY4) [68,69]. The exact role of the *HES1* protein in the adult pancreas has yet to be determined. It is expressed in the duct and centroacinar cells in the gland and is involved in the differentiation of ductal and acinar cells under normal and inflammatory conditions [37].

A key challenge in pancreatic cancer research is differentiating tumor-driven inflammation from systemic inflammatory responses associated with benign pancreatic disorders, such as acute and chronic pancreatitis. While our study demonstrates a strong correlation between *CK19*, *NANOG*, and *INS* mRNA expression and systemic inflammatory markers, it remains unclear whether these alterations are exclusive to PDAC or reflect a broader inflammation-associated gene-expression changes. Further studies will provide critical insights into their effectiveness to discriminate malignancy from inflammation-associated gene-expression changes.

The limitation of our study is the lack of heterogeneity among cancer patients, as only those with advanced-stage disease (metastatic or locally advanced disease) were included. Furthermore, we acknowledge the presence of considerable inter-individual variability and small differences in the analysis results. While these variations pose challenges in biomarker standardization, they also highlight the complexity of pancreatic tumor biology and underline the need for larger, prospective studies to validate these findings.

Furthermore, the identification of stage-specific biomarkers and their integration with established markers, such as Ca 19-9, may enhance the sensitivity and specificity of early pancreatic cancer detection. Given the limitations of Ca 19-9, including its elevation in benign inflammatory conditions and its low sensitivity for early-stage PDAC, incorporating mRNA expression patterns of *CK19*, *NANOG*, and *INS* into a multi-marker panel could improve diagnostic accuracy. Future studies should focus on determining whether the expression of these genes differs between localized and metastatic disease, thereby identifying potential markers that emerge at the earliest stages of malignancy. Moreover, longitudinal analyses of mRNA expression before and after treatment could help assess if changes in these markers correlate with therapeutic response, disease progression, or recurrence risk. Prospective trials focusing on mRNA expression in high-risk populations, such as individuals with hereditary predispositions or long-standing chronic pancreatitis, may further elucidate the clinical use of those biomarkers for early detection.

The absence of long-term survival data in our study represents another limitation. Evaluating how these gene-expression patterns correlate with clinical characteristics, including disease progression, would provide a better understanding of their prognostic significance. Moreover, the absence of publicly available dataset containing expression profiles of the genes analyzed in this study within an independent cohort of PDAC or PNET patients, further underscores the need for external validation. Establishing a well-characterized validation cohort is crucial for validation of our findings and ensuring the clinical application.

Despite these limitations, our study is the first to comprehensively assess the mRNA expression of stem cells and progenitor genes across a spectrum of neoplastic pancreatic disorders. These findings provide a strong foundation for future research aimed to focus on molecular diagnostic and prognostic tools in pancreatic cancer, ultimately improving early detection and risk stratification.

## 5. Conclusions

The mRNA expression of selected stem and progenitor genes (*NANOG*, *INS*, *CK19*) in peripheral-blood mononuclear cells has potential as a valuable marker for pancreatic cancer, with a notable correlation with coexisting inflammation. These findings underscore the importance of studying mRNA expression in peripheral blood to enhance our understanding of pancreatic neoplasms. This approach may contribute to improving detection and enabling more personalized, non-invasive strategies for managing these aggressive diseases.

## Figures and Tables

**Table 1:** Characteristics of PDAC and PNET patients.

	PDAC *N* = 37	PNETs *N* = 12	Controls *N* = 34
Age (years)	64 (57–69)	51.5 (38–58.5) *	61 (49–73)
Height (cm)	168 (160–175)	171.5 (165.5–178.5)	166.5 (162–173)
Weight (kg)	65 (55–74) *	70 (60–75.5)	75 (63–80)
Female/Male	23/14	5/7	25/9
BMI (kg/m^2^)	22.5 (20–25.8) *	23.3 (20.7–25.7)	25 (23.4–28.6)
WBC (K/μL)	8.2 (6.5–10.2) *	6.47 (4.7–7.4)	6.2 (5.2–7.3)
Plt (K/μL)	233 (167–276)	220 (198–327)	229.5 (185–267)
Ery (mln/μL)	4.3 (3.7–4.7) *	4.5 (4.3–4.9)	4.7 (4.3–4.9)
CRP (mg/L)	11 (3.46–74) *	2 (1–4.7)	2.5 (1–5)
Ca 19-9	482.2 (114.8–5890) *	6.1 (3.5–9.0)	4.2 (2–8.9)

Data are presented as medians with interquartile ranges. * *p* < 0.05 vs. healthy controls, Mann–Whitney U test.

**Table 2:** Gene-expression levels of six genes (*POU5F1*, *NANOG*, *HES1*, *PDX*, *INS*, *CK19*) in the control (N = 34), PNETS (N = 12), and PDAC (N = 34) groups.

	Control N = 34	PNETs N = 12	PDAC N = 34
*POU5F1*	0.002 (0.0005–0.006)	0.0009 (0.0002–0.004)	0.003 (0.001–0.01)
*NANOG*	0.0003 (0.00005–0.004)	0.0008 (0.00008–0.003)	0.002 (0.0003–0.005) *
*HES1*	0.00011 (0.00003–0.0006)	0.00009 (1.3 × 10^−6^–0.0009)	0.0001 (0.0003–0.0003)
*PDX*	0.00003 (6.5 × 10^−6^–0.0001)	0.00003 (3.7 × 10^−6^–0.0005)	0.00008 (0.00002–0.0002
*INS*	0.00008 (2.7 × 10^−6^–0.0005)	0.0002 (1.8 × 10^−6^–0.005)	0.0004 (0.00004–0.002) *
*CK19*	0.000022 (2.9 × 10^−6^–0.0002)	0.00008 (9.7 × 10^−6^–0.004)	0.0003 (0.00007–0.001) *

Data are presented as medians with interquartile ranges. * *p* < 0.05 vs. healthy controls, Mann–Whitney U test.

**Table 3:** Comparison of basic clinical characteristics and mRNA expression in patients with locally advanced and metastatic pancreatic cancer.

	Locally Advanced N = 23	Metastatic N = 14
Age	61 (55–66) *	67.7 (63–74) *
*POU5F1*	0.003 (0.0007–0.009)	0.003 (0.001–0.03)
*NANOG*	0.002 (0.0002–0.005)	0.002 (0.0004–0.006)
*HES1*	0.0001 (0.00005–0.0003)	0.00009 (0.00003–0.0005)
*PDX*	0.00008 (0.00001–0.0001)	0.00009 (0.00005–0.0006)
*INS*	0.0004 (0.00004–0.002)	0.0007 (0.00004–0.004)
*CK19*	0.0002 (0.00008–0.001)	0.0003 (0.00001–0.002)

Data are presented as medians with interquartile ranges. * *p* < 0.05 vs. healthy controls, Mann–Whitney U test.

## Data Availability

The original data presented in the study are openly available in FigShare repository at https://doi.org/10.6084/m9.figshare.27921831.v1 accessed on 25 March 2025.

## Supplementary Materials

The following supporting information can be downloaded at: https://www.mdpi.com/article/10.3390/cimb47040236/s1.
