# Supplementary material for: An Analysis of the mRNA Expression of Peripheral-Blood Stem and Progenitor Cell Markers in Pancreatic Neoplastic Disorders
Source: Curr Issues Mol Biol. 2025 Mar 28;47(4):236. doi: 10.3390/cimb47040236 (PMC12025646; doi:10.3390/cimb47040236)
Supplement: Supplementary file 1 [file cimb-47-00236-s001.zip › cimb-3506444-supplementary.pdf]

Supplementary Materials.

Table S1. Correlations between mRNA gene expression and clinical and biochemical characteristics in patients with PDAC.

	WBC	PLT	CRP	ERY	Age	Weight	BMI
POU5F1	0.14	−0.15	0.07	0.03	0.02	−0.07	−0.1
NANOG	0.17	−0.06	0.03	0.01	−0.06	−0.1	−0.16
CK19	0.51 *	0.06	0.34 *	−0.16	0.12	−0.06	−0.04
PDX	0.25	−0.16	0.06	0.05	−0.09	−0.25	−0.2
INS	0.32	−0.02	0.1	−0.07	0.05	−0.1	−0.12
HES1	−0.16	−0.11	0.24	−0.21	0.27	0.01	0.23

The data are presented as values of the Spearman rank correlation coefficient. * $p < 0.05$.

Table S2. Correlations between mRNA gene expression and clinical and biochemical characteristics in patients with PNET.

	WBC	PLT	CRP	ERY	Age	Weight	BMI
POU5F1	0.14	−0.07	−0.06	−0.12	−0.12	−0.37	−0.21
NANOG	−0.3	−0.54	−0.09	0.22	−0.24	−0.27	−0.12
CK19	−0.14	−0.2	0.06	0.4	−0.14	−0.06	0.19
PDX	0.05	−0.5	−0.08	0.31	−0.28	−0.45	−0.24
INS	−0.04	−0.55	0.17	0.51	−0.27	−0.06	0.1
HES1	0.77 *	0.16	−0.01	−0.15	0.02	−0.16	0.01

The data are presented as values of the Spearman rank correlation coefficient. * $p < 0.05$.
